# The gut mycobiome: The overlooked constituent of clinical outcomes and treatment complications in patients with cancer and other immunosuppressive conditions

**DOI:** 10.1371/journal.ppat.1008353

**Published:** 2020-04-02

**Authors:** Jessica R. Galloway-Peña, Dimitrios P. Kontoyiannis

**Affiliations:** 1 Department of Genomic Medicine, The University of Texas MD Anderson Cancer Center, Houston, Texas, United States of America; 2 Department of Infectious Diseases, Infection Control, and Employee Health, The University of Texas MD Anderson Cancer Center, Houston, Texas, United States of America; Vallabhbhai Patel Chest Institute, INDIA

Extensive efforts have focused on investigating the contributions of the intestinal microbiome to health and disease, including immunomodulation [1, 2]. While the term “microbiome” technically refers to microorganisms including bacteria, fungi, viruses, protozoa, and parasites, the majority of studies focus on the bacteriome [3]. Although the bacteriome constitutes >99% of the microbiome [4] (which is potentially the reason most studies focus on the bacteriome), it is irrefutable that the commensal fungi, or the “mycobiome,” alongside the other microorganisms, coexist and interact ways that can be beneficial or detrimental to the host [5–7]. Emerging research has focused on how the bacteriome relates to gastrointestinal (GI) disorders, cancer therapy–related toxicities, and stem-cell transplantation outcomes; including correlations with infection, graft-versus-host disease (GvHD), tumorigenesis, cancer relapse, and mortality [8–11]. Thus far, there has been a lack of dedicated research focusing on the influence of mycobiome-associated immunomodulation in patients with cancer and other states of immunosuppression. Herein, by focusing on the gut ecosystem, we discuss the role of fungi in various patient populations, the importance of bacterial-fungal dysbiosis, and offer ideas for future investigations regarding the role of mycobiome.

## Current implications of gut fungi in patients with cancer or critical illness

Fungal diversity and density are low in healthy subjects [7], although the factors for colonization resistance against fungi in the gut are inadequately understood. It has been long known that commensal bacteria limit fungal colonization via activation of mucosal innate immunity by bacterial derived metabolites [12], while antibacterial agents can predispose individuals to *Candida albicans* colonization and infections [13]. For example, antibiotic-induced dysbiosis of intestinal microbes, such as *Bacteriodes spp*., was linked to a reduction in the cathelicidin antimicrobial peptide (CRAMP), which resulted in the outgrowth of intestinal *Candida* spp. [12]. Recently, it was found that an antibiotic-induced reduction in the levels of bacterial derived short-chain fatty acids (SCFAs) in the cecum enhanced GI colonization of *C*. *albicans* [14].

Antibiotics, however, are not the only factor that can potentially result in increased fungal burden in the gut. In addition to the known effects of proton pump inhibitors (PPIs) as promoters of *Candida* gut colonization [15], it has been shown that high-intensity chemotherapy results in reduced diversity of the GI microbiota, reduction of anaerobes [16], and a shift in the Firmicutes to Bacteriodetes ratio [17]. Given that decreases of anaerobic bacteria have been shown to promote *Candida* overgrowth [18], and Bacteroidetes have been shown to be negatively associated with fungi [19, 20], it stands to reason that the gut mycobiome is altered during cytotoxic chemotherapy.

In addition to the known fact that *Candida* colonization precedes *Candida* invasion in the bloodstream [21], enrichment of the fungal consortium in the gut could also affect cancer treatment-related complications and oncological outcomes. It was previously shown that prolonged administration of fluconazole for 75 days after hematopoietic stem cell transplantation (HSCT) was not only associated with protection against invasive candidiasis and *Candida*-related death, but also with decreased gut GvHD [22]. Indeed, *Candida*-colonized patients have significantly higher incidence of severe GvHD [23]. C-type lectin receptors (such as Dectin-1 and -2) of dendritic cells recognize fungal cell wall polysaccharides, which trigger protective antifungal T helper 17 cell (Th17) responses in the GI mucosa [24]. In fact, the induction of Th17/interleukin-23 (IL-23) responses via activation of pattern recognition receptors by *Candida* has been suggested as a potential mechanism of GvHD pathophysiology [25].

The alterations in gut bacterial metabolites in the setting of antibiotics, chemotherapy, and HSCT might have indirect effects on fungal fitness and morphogenesis. It has been shown that antibiotics with activity against anaerobic organisms can reduce gut SCFA levels [26] and that low fecal butyrate and propionate levels correlate with decreased microbial diversity and higher incidence of GvHD post HSCT [27]. Interestingly, SCFAs have been demonstrated to induce transcriptional changes in *C*. *albicans* [28] and butyric acid has been found to inhibit yeast–hyphal transition [29]. Furthermore, it is possible antibiotics may have an indirect role on adverse GvHD outcomes by affecting *Candida* physiology, such as promoting yeast-to-hyphae transition [30].

In terms of tumorogenesis, the mycobiome has been implicated in the pathogenesis of colon adenomas [31] and, most recently, pancreatic ductal adenocarcinoma [32]. It was shown that fungi migrate from the gut to the pancreas and that pancreatic tumors are infiltrated by *Malassezia* spp. Removal of the mycobiome was protective against tumor growth. Mechanistically, fungi promoted the progression of pancreatic cancer by inducing the complement cascade via activation by mannose binding lectin. Similarly, researchers have also recently shown increases in Malasseziomycetes and decreases in Saccharomycetes in patients with colorectal cancer, but no mechanism has been proposed [33].

## The mycobiome and chronic inflammatory bowel disorders

In addition to the cancer and critically ill setting, the gut mycobiome has also been implicated in inflammatory GI disorders, to include Crohn disease and ulcerative colitis. It was first observed that patients with inflammatory bowel disorders (IBDs) had a higher GI colonization rate by *C*. *albicans* compared to healthy individuals [34]. Furthermore, Sokol and colleagues observed an imbalance in the Basidiomycota to Ascomycota ratio in IBD compared to healthy subjects [35]. Further mechanistic studies implicated the mycobiome as a key contributor to initiation of inflammation and pathogenesis of IBD, where dectin-1–deficient mice had more severe IBD symptoms and colonization by pathogenic fungi [36]. It was also shown that *C*. *tropicalis* could exacerbate colitis severity in dectin-1–deficient mice. However, the association of antifungal selection pressure to the constitution of the gut mycobiome and its direct or indirect consequences to the underlying GI pathology and microbiome are rather complex. For example, prolonged treatment with fluconazole led to decreased levels of *Candida* gut colonization at the expense of increased levels of gut colonization by opportunistic molds, such as *Aspergillus amstelodami*, resulting in elevated colitis severity [37]. Interestingly, susceptibility to colitis occurring in the presence of commensal bacteria eradication was revered by colonization with either *C*. *albicans* or *Saccharomyces cerevisiae* [6]. This shows that in the appropriate context, fungi can also confer protection against mucosal injury by tuning immune response.

### Fungal–bacterial interactions to consider in the patient with cancer or GI disorder, or critically ill patient

Many bacterial–fungal interactions that have been reported to influence the colonization and pathogenesis of both kingdoms (Table 1). However, most studies were derived from in vitro experiments or murine models that have a mono-microbial view of alterations in fungal biology as a result of interactions with bacteria. These interactions have been shown to provide synergy in commensalism, as in the case of *C*. *albicans* with enterococci [38], or are mutually antagonistic, such as the case between *C*. *albicans* and *Pseudomonas aeruginosa* [39] (Table 1). Interestingly, fecal microbiota transplantation (FMT) efficacy was reduced in patients with *Clostridioides difficile* colitis who had dominance of *Candida* in the gut [40]. One possible reason why patients with *C*. *difficile* and co-colonized with *Candida* species may not respond to FMT is that *C*. *albicans* has also been shown to affect gut bacterial reconstitution or recolonization after antibiotic administration [19]. Given that FMT has become an attractive treatment strategy, not only for *C*. *difficile* infection or GI disorders but also to mitigate other treatment-related toxicities such as GvHD, immune checkpoint inhibitor–associated colitis, and antibiotic resistant infection, one must consider the fungal contribution to the effectiveness of this strategy [41–44].

**Table 1:** Important fungal–bacterial interactions altering pathogenesis.

Fungi	Bacteria	Relationship	Model	Observation	Reference
***Candida albicans***	*Enterococcus* spp.	Synergy	Germ-free and antibiotic-perturbed mice	Enterococcal species are found to dominate the gastrointestinal microbiome following the introduction of *C*. *albicans*	[38]
		Antagonistic	*C*. *elegans* coinfection model	*E*. *faecalis* can inhibit *C*. *albicans* hyphal morphogenesis and virulence	[38]
***C***. ***albicans***	*Pseudomonas aeruginosa*	Antagonistic	In vitro models	*P*. *aeruginosa* lipopolysaccharide inhibits *C*. *albicans* biofilm and hyphal development	[39]
			In vitro models	*P*. *aeruginosa* excretes quorum-sensing molecules and quinolone signals, which repress hyphal and biofilm formation	[39]
			In vitro models	*C*. *albicans* secretes farnesol, which down-regulates the expression of *P*. *aeruginosa* virulence factors through modulation of the *Pseudomonas* quinolone signal system	[39]
			In vitro models	*C*. *albicans* inhibits the production of cytotoxic exotoxin A and pyoverdine	[39]
			Neutropenic co-colonized mice	Mice colonized with both *P*. *aeruginosa* and *C*. *albicans* had significantly lower mortality compared to those colonized with *P*. *aeruginosa* alone	[39]
***C***. ***albicans***	*Clostridium* spp.	Synergy	In vitro models	*C*. *albicans* coculture promotes *C*. *difficile* and *C*. *perfringens* growth in aerobic conditions	[63]
			*C*. *difficile* mouse model	Oral *Candida* administration worsens *C*. *difficile* severity	[64]
		Antagonistic	In vitro models	p-cresol, produced by *C*. *difficile*, inhibits hyphal formation and virulence of *C*. *albicans*	[63]
			*C*. *difficile* mouse model	*C*. *albicans* reduces *C*. *difficile* growth and *C*. *difficile*–related mortality, which appears dependent on the alterations that *Candida* induces on the gut bacteriome composition	[40]

## Fungal implications for immunomodulation

There are data analogous to the bacteriome which suggest the immunomodulatory role of fungi colonizing the GI tract in both innate and adaptive immunity [24]. It is well characterized that gut colonization by *Candida* or other fungi elicits Th17 and Th1 responses [45, 46]. In fact, among 30 taxa of the human mycobiome, *C*. *albicans* is the major inducer of systemic Th17 cells [47]. Additionally, *Candida*-specific Foxp3^+^ regulatory T (Treg) cells, which are implicated in the maintenance of mucosal immune homeostasis, have been detected in the peripheral blood of healthy individuals [48]. Depending on the morphogenetic state of *S*. *cerevisiae*, both subsets from human CD4^+^ T cells can be induced; thus, *S*. *cerevisiae* yeasts induce Th1 CD4 differentiation, while *S*. *cerevisiae* spores promote Th17 CD4 expansion. These differential effects of fungi on T-cell responses appear to be dependent on the influence of fungal mannans on dendritic cells [49]. Moreover, as discussed above, inoculation with *S*. *cerevisiae* and *C*. *albicans* were sufficient to alleviate the severe colitis as well as reduced levels of protective CD8^+^ T cells in antibiotic-treated mice infected with influenza virus [6].

Emerging data suggest that gut microbes may impact antitumor immunity during immunotherapy by priming innate effectors and the adaptive immune responses, inducing cytokine production by antigen-presenting cells or lymphocytes [32]. In the aforementioned data, gut mycobiota (specifically *Malassezia* species) are implicated in the pathogenesis of pancreatic adenocarcinoma by promoting pancreatic inflammation through the complement cascade [32]. Interestingly, several in vitro studies using myeloid or keratinocyte cell lines have shown that stimulation with *Malassezia* leads to the induction of mainly proinflammatory cytokines and chemokines [50]. Given the evidence for the immunomodulatory role of the gut mycobiota, it is important to consider the effects perturbation of the gut fungi may cause on human health and various disease states, including possibly sites distant from the gut, such as lungs [47] and central nervous system [51].

The immunomodulatory role of fungi may not only have implications for chemotherapeutic/immunotherapy response and leukemia persistence, but also for infectious complications. It has been shown that in scenarios where antibiotics promote *Candida* intestinal domination, genetic changes occur that lead to increased fitness of *C*. *albicans* in the gut [52]. Interestingly, this gut-adapted *C*. *albicans* confers increased protection against systemic fungal and bacterial pathogens [52], likely due to the induction of systemic adaptive Th17 responses [46]. *C*. *albicans* and *S*. *cerevisiae* were both shown to be capable of stimulating innate immunological memory in myeloid cells [53]. Interestingly, mice treated with β‐1,3‐glucan or chitin were protected from a *C*. *albicans* challenge, suggesting a mechanism by which fungi can train mucosal or circulating monocytes [54].

## Technical considerations for mycobiome studies

The mycobiome field is in its infancy, and thus many technical challenges need to be considered when performing these studies. First, compared to bacteria and viruses, the mycobiome comprises a relatively minor component of the overall microbiome [7]. Many commonly used fecal genomic DNA extraction protocols are tailored for extracting bacterial genomic DNA and are often imperfect for extracting fungal genomic DNA in regard to bead size for mechanical lysis, enzymatic lysis buffers, and neutralizing or stabilizing agents [55]. Moreover, different extraction kits favor particular fungal species, are biased against others, and are prone to contamination [56]. Thus, one must carefully consider DNA extraction methods based on whether the study is mycobiome specific, or if one needs to combine bacterial and fungal microbiota analyses.

Most problems, however, lie in the lack of standardized methods for characterization of the mycobiota. When comparing amplicon sequencing, ITS1, ITS2, 18S, and 28S rRNA give slightly different results [56]. The 18S rRNA typically outperforms other markers in its ability to amplify and discriminate different species; however, because fungal rRNA copy numbers vary, there is a strong bias towards fungi with more copies. On the other hand, although the internal transcribed spacer (ITS) region represents the formal fungal barcode, it sometimes provides insufficient resolution to distinguish species. ITS primers show both amplification and sequencing biases related to the variable length of the product [56]. The length of the ITS1 and ITS2 markers vary from 50 bp to several kb. Incorrect mapping, and thus classification, leads to the inclusion of false positives or exclusion of valid operational taxonomic units (OTUs). Microbiome studies rely on well-curated reference databases in order to provide accurate taxonomic assignments of OTUs. Unfortunately, public repositories contain a high percentage of fungal sequences that are incomplete or even incorrectly annotated [57, 58]. Moreover, in regard to shotgun metagenomics, the number of annotated fungal genome sequences available in reference databases are sparse compared to the number of bacterial genome sequences available. Thus, for the mycobiome field to move forward, it would be critical to expand fungal sequencing efforts and improve fungal phylogenetics and taxonomic classification.

Another critical topic to consider in research moving forward is the potential for using mycobiome components as rapid diagnostic markers. Despite their promise, the diagnostic use of fungal biomarkers, such as galactomannan and beta glucan, are fraught with problems even in high-risk populations, such as acute myeloid leukemia (AML) and HSCT patients [59]. In contrast, mycobiome testing offers the promise of a holistic assessment of the fungal community in a particular site. As with all molecular-based clinical diagnostics in mycology, technical challenges include the sheer spectrum and number of fungi needed to be identified in immunocompromised patients, universal methods for preparing sample templates considering fungal morphology variability, lack of consistency in nomenclature, and the limitations of commercial platforms panels, reference libraries, and databases [60].

## Conclusions

Although the impact of the microbiome in health and disease has been established, concurrent analysis of the bacterial/fungal consortium and its balance have been understudied [61]. This knowledge gap may be in part due to technical limitations within the metagenomic field; however, one can imagine that there is a vast number of cross-kingdom interactions that are important in the human host. To date, a small number of fungal-bacterial relationships have been studied in vitro or in model systems, but often this “one bacteria, one fungi” experimentation strips these insights of their complexity and the nuances of interactions in the setting of polymicrobial communities. This multifaceted *trans*-kingdom interplay is not only affected by the specific members that are present but also the complex immunological millieu of the human host. Fungal–bacterial combinations that may be neutral or advantageous in the human host may be detrimental in an immunocompromised patient. Thus, improved approaches in understanding the mycobiome are vital in order to provide a foundation for personalized medicine in the patient with cancer. A deeper comprehension of the fungal–bacterial–immunocompromised host triad may allow for identification of high-risk patients and improved treatment strategies [62]. We believe mycobiome is an underexploited field of investigation in the cancer field, and many fascinating research questions remain unanswered in both patients with hematologic cancer (Box 1) and other patients with chronic immunosuppressive conditions (e.g., recipients of solid transplant, chronic autoimmune disorders, and critically ill patients in the intensive care unit (ICU), where systematic mycobiome research is currently limited.

**Box 1. Mycobiome-related questions for future investigation in patients with malignancy.**

1. What is the impact of mycobiome maintenance on a diverse, "healthy" microbiome following cancer therapy (or vice versa)?
2. What is the metabolic role of fungi within the gut microbiome? Are there any relationships between *Candida* dominance and microbial abundances of vancomycin-resistant enterococci (VRE), *Pseudomonas*, *Clostridia*, or streptococci in the patient with cancer? If so, can the mycobiome predict subsequent bacterial infections originating from domination in the gut?
3. Given that some chemotherapeutic agents have specific anti-*Candida* activities, are there any relationships between the mycobiome profiles and specific chemotherapies? Are mycobiome changes that occur as a result of chemotherapy GI specific, or do they occur elsewhere (e.g., nares, oral, lung)?
4. Are there any relationships between the baseline mycobiome with other clinical factors such as age, ethnicity, geographic, and leukemia cytogenetics? Are there any relationships of the mycobiome and key polymorphisms in pattern recognition receptors or other proteins involved in immune function (e.g., Toll-like receptors TLR2 and TLR4, dectin-1, NOD2 [nucleotide-binding oligomerization domain-containing protein 2], IL-17A, IL-22, or Foxp3^+^)?
5. Are mycobiome changes related to probability and duration of remission?
6. Can microbiome manipulation with pro- or prebiotics result in a better mycobiome profile following cancer therapy–induced dysbiosis?

## References

[1] Galloway-Pena J, Brumlow C, Shelburne S. Impact of the Microbiota on Bacterial Infections during Cancer Treatment. Trends Microbiol. 2017;25(12):992–1004. 10.1016/j.tim.2017.06.006 28728967

[2] Gopalakrishnan V, Helmink BA, Spencer CN, Reuben A, Wargo JA. The Influence of the Gut Microbiome on Cancer, Immunity, and Cancer Immunotherapy. Cancer Cell. 2018;33(4):570–80. 10.1016/j.ccell.2018.03.015 29634945 PMC6529202

[3] El-Jurdi N, Ghannoum MA. The Mycobiome: Impact on Health and Disease States. Microbiol Spectr. 2017;5(3). 10.1128/microbiolspec.FUNK-0045-2016 PMC11687503 28597821

[4] Qin J, Li R, Raes J, Arumugam M, Burgdorf KS, Manichanh C, et al A human gut microbial gene catalogue established by metagenomic sequencing. Nature. 2010;464(7285):59–65. 10.1038/nature08821 20203603 PMC3779803

[5] Hoarau G, Mukherjee PK, Gower-Rousseau C, Hager C, Chandra J, Retuerto MA, et al Bacteriome and Mycobiome Interactions Underscore Microbial Dysbiosis in Familial Crohn's Disease. MBio. 2016;7(5). 10.1128/mBio.01250-16 PMC5030358 27651359

[6] Jiang TT, Shao TY, Ang WXG, Kinder JM, Turner LH, Pham G, et al Commensal Fungi Recapitulate the Protective Benefits of Intestinal Bacteria. Cell Host Microbe. 2017;22(6):809–16 e4. 10.1016/j.chom.2017.10.013 29174402 PMC5730478

[7] Nash AK, Auchtung TA, Wong MC, Smith DP, Gesell JR, Ross MC, et al The gut mycobiome of the Human Microbiome Project healthy cohort. Microbiome. 2017;5(1):153 10.1186/s40168-017-0373-4 29178920 PMC5702186

[8] Galloway-Pena JR, Shi Y, Peterson CB, Sahasrabhojane P, Gopalakrishnan V, Brumlow CE, et al Gut Microbiome Signatures are Predictive of Infectious Risk Following Induction Therapy for Acute Myeloid Leukemia. Clin Infect Dis. 2019 10.1093/cid/ciz777 PMC7312220 31436833

[9] Shono Y, van den Brink MRM. Gut microbiota injury in allogeneic haematopoietic stem cell transplantation. Nat Rev Cancer. 2018;18(5):283–95. 10.1038/nrc.2018.10 29449660 PMC7485905

[10] Clemente JC, Manasson J, Scher JU. The role of the gut microbiome in systemic inflammatory disease. BMJ. 2018;360:j5145 10.1136/bmj.j5145 29311119 PMC6889978

[11] Picardo SL, Coburn B, Hansen AR. The microbiome and cancer for clinicians. Crit Rev Oncol Hematol. 2019;141:1–12. 10.1016/j.critrevonc.2019.06.004 31202124

[12] Fan D, Coughlin LA, Neubauer MM, Kim J, Kim MS, Zhan X, et al Activation of HIF-1alpha and LL-37 by commensal bacteria inhibits *Candida albicans* colonization. Nat Med. 2015;21(7):808–14. 10.1038/nm.3871 26053625 PMC4496259

[13] Samonis G, Gikas A, Anaissie EJ, Vrenzos G, Maraki S, Tselentis Y, et al Prospective evaluation of effects of broad-spectrum antibiotics on gastrointestinal yeast colonization of humans. Antimicrob Agents Chemother. 1993;37(1):51–3. 10.1128/aac.37.1.51 8431017 PMC187603

[14] Guinan J, Wang S, Hazbun TR, Yadav H, Thangamani S. Antibiotic-induced decreases in the levels of microbial-derived short-chain fatty acids correlate with increased gastrointestinal colonization of *Candida albicans*. Sci Rep. 2019;9(1):8872 10.1038/s41598-019-45467-7 31222159 PMC6586901

[15] Jacobs DM, Beyda ND, Asuphon O, Alam MJ, Garey KW. Host factors and clinical outcomes of *Candida colonization* in critically ill patients. Mycopathologia. 2015;179(1–2):87–93. 10.1007/s11046-014-9809-6 25173925

[16] van Vliet MJ, Tissing WJ, Dun CA, Meessen NE, Kamps WA, de Bont ES, et al Chemotherapy treatment in pediatric patients with acute myeloid leukemia receiving antimicrobial prophylaxis leads to a relative increase of colonization with potentially pathogenic bacteria in the gut. Clin Infect Dis. 2009;49(2):262–70. 10.1086/599346 19514856

[17] Montassier E, Batard E, Massart S, Gastinne T, Carton T, Caillon J, et al 16S rRNA gene pyrosequencing reveals shift in patient faecal microbiota during high-dose chemotherapy as conditioning regimen for bone marrow transplantation. Microb Ecol. 2014;67(3):690–9. 10.1007/s00248-013-0355-4 24402367

[18] Charlet R, Bortolus C, Barbet M, Sendid B, Jawhara S. A decrease in anaerobic bacteria promotes *Candida glabrata* overgrowth while beta-glucan treatment restores the gut microbiota and attenuates colitis. Gut Pathog. 2018;10:50 10.1186/s13099-018-0277-2 30524506 PMC6276212

[19] Mason KL, Erb Downward JR, Mason KD, Falkowski NR, Eaton KA, Kao JY, et al *Candida albicans* and bacterial microbiota interactions in the cecum during recolonization following broad-spectrum antibiotic therapy. Infect Immun. 2012;80(10):3371–80. 10.1128/IAI.00449-12 22778094 PMC3457555

[20] Neville BA, d'Enfert C, Bougnoux ME. *Candida albicans* commensalism in the gastrointestinal tract. FEMS Yeast Res. 2015;15(7). 10.1093/femsyr/fov081 26347504

[21] Dalle F, Lafon I, L'Ollivier C, Ferrant E, Sicard P, Labruere C, et al A prospective analysis of the genotypic diversity and dynamics of the *Candida albicans* colonizing flora in neutropenic patients with de novo acute leukemia. Haematologica. 2008;93(4):581–7. 10.3324/haematol.11882 18322258

[22] Marr KA, Seidel K, Slavin MA, Bowden RA, Schoch HG, Flowers ME, et al Prolonged fluconazole prophylaxis is associated with persistent protection against candidiasis-related death in allogeneic marrow transplant recipients: long-term follow-up of a randomized, placebo-controlled trial. Blood. 2000;96(6):2055–61. 10979947

[23] van der Velden WJ, Plantinga TS, Feuth T, Donnelly JP, Netea MG, Blijlevens NM. The incidence of acute graft-versus-host disease increases with *Candida* colonization depending the dectin-1 gene status. Clin Immunol. 2010;136(2):302–6. 10.1016/j.clim.2010.04.007 20452827

[24] Rizzetto L, De Filippo C, Cavalieri D. Richness and diversity of mammalian fungal communities shape innate and adaptive immunity in health and disease. Eur J Immunol. 2014;44(11):3166–81. 10.1002/eji.201344403 25257052

[25] van der Velden WJ, Netea MG, de Haan AF, Huls GA, Donnelly JP, Blijlevens NM. Role of the mycobiome in human acute graft-versus-host disease. Biol Blood Marrow Transplant. 2013;19(2):329–32. 10.1016/j.bbmt.2012.11.008 23160005

[26] Romick-Rosendale LE, Haslam DB, Lane A, Denson L, Lake K, Wilkey A, et al Antibiotic Exposure and Reduced Short Chain Fatty Acid Production after Hematopoietic Stem Cell Transplant. Biol Blood Marrow Transplant. 2018;24(12):2418–24. 10.1016/j.bbmt.2018.07.030 30055351

[27] Mathewson ND, Jenq R, Mathew AV, Koenigsknecht M, Hanash A, Toubai T, et al Gut microbiome-derived metabolites modulate intestinal epithelial cell damage and mitigate graft-versus-host disease. Nat Immunol. 2016;17(5):505–13. 10.1038/ni.3400 26998764 PMC4836986

[28] Cottier F, Tan AS, Chen J, Lum J, Zolezzi F, Poidinger M, et al The transcriptional stress response of *Candida albicans* to weak organic acids. G3 (Bethesda). 2015;5(4):497–505. 10.1534/g3.114.015941 25636313 PMC4390566

[29] Shareck J, Belhumeur P. Modulation of morphogenesis in *Candida albicans* by various small molecules. Eukaryot Cell. 2011;10(8):1004–12. 10.1128/EC.05030-11 21642508 PMC3165445

[30] McCool L, Mai H, Essmann M, Larsen B. Tetracycline effects on Candida albicans virulence factors. Infect Dis Obstet Gynecol. 2008;2008:493508 10.1155/2008/493508 18528520 PMC2408679

[31] Luan C, Xie L, Yang X, Miao H, Lv N, Zhang R, et al Dysbiosis of fungal microbiota in the intestinal mucosa of patients with colorectal adenomas. Sci Rep. 2015;5:7980 10.1038/srep07980 25613490 PMC4648387

[32] Aykut B, Pushalkar S, Chen R, Li Q, Abengozar R, Kim JI, et al The fungal mycobiome promotes pancreatic oncogenesis via activation of MBL. Nature. 2019;574(7777):264–7. 10.1038/s41586-019-1608-2 31578522 PMC6858566

[33] Coker OO, Nakatsu G, Dai RZ, Wu WKK, Wong SH, Ng SC, et al Enteric fungal microbiota dysbiosis and ecological alterations in colorectal cancer. Gut. 2019;68(4):654–62. 10.1136/gutjnl-2018-317178 30472682 PMC6580778

[34] Standaert-Vitse A, Sendid B, Joossens M, Francois N, Vandewalle-El Khoury P, Branche J, et al *Candida albicans* colonization and ASCA in familial Crohn's disease. Am J Gastroenterol. 2009;104(7):1745–53. 10.1038/ajg.2009.225 19471251

[35] Sokol H, Leducq V, Aschard H, Pham HP, Jegou S, Landman C, et al Fungal microbiota dysbiosis in IBD. Gut. 2017;66(6):1039–48. 10.1136/gutjnl-2015-310746 26843508 PMC5532459

[36] Iliev ID, Funari VA, Taylor KD, Nguyen Q, Reyes CN, Strom SP, et al Interactions between commensal fungi and the C-type lectin receptor Dectin-1 influence colitis. Science. 2012;336(6086):1314–7. 10.1126/science.1221789 22674328 PMC3432565

[37] Wheeler ML, Limon JJ, Bar AS, Leal CA, Gargus M, Tang J, et al Immunological Consequences of Intestinal Fungal Dysbiosis. Cell Host Microbe. 2016;19(6):865–73. 10.1016/j.chom.2016.05.003 27237365 PMC4900921

[38] Garsin DA, Lorenz MC. *Candida albicans* and *Enterococcus faecalis* in the gut: synergy in commensalism? Gut Microbes. 2013;4(5):409–15. 10.4161/gmic.26040 23941906 PMC3839987

[39] Fourie R, Pohl CH. Beyond Antagonism: The Interaction Between *Candida* Species and *Pseudomonas aeruginosa*. J Fungi (Basel). 2019;5(2). 10.3390/jof5020034 PMC6617365 31010211

[40] Stewart D, Romo JA, Lamendella R, Kumamoto CA. The role of fungi in *C*. *difficile* infection: An underappreciated transkingdom interaction. Fungal Genet Biol. 2019;129:1–6. 10.1016/j.fgb.2019.04.007 30978391 PMC6642687

[41] Battipaglia G, Malard F, Rubio MT, Ruggeri A, Mamez AC, Brissot E, et al Fecal microbiota transplantation before or after allogeneic hematopoietic transplantation in patients with hematologic malignancies carrying multidrug-resistance bacteria. Haematologica. 2019;104(8):1682–8. 10.3324/haematol.2018.198549 30733264 PMC6669143

[42] Kakihana K, Fujioka Y, Suda W, Najima Y, Kuwata G, Sasajima S, et al Fecal microbiota transplantation for patients with steroid-resistant acute graft-versus-host disease of the gut. Blood. 2016;128(16):2083–8. 10.1182/blood-2016-05-717652 27461930 PMC5085256

[43] Webb BJ, Brunner A, Ford CD, Gazdik MA, Petersen FB, Hoda D. Fecal microbiota transplantation for recurrent *Clostridium difficile* infection in hematopoietic stem cell transplant recipients. Transpl Infect Dis. 2016;18(4):628–33. 10.1111/tid.12550 27214585

[44] Wang Y, Wiesnoski DH, Helmink BA, Gopalakrishnan V, Choi K, DuPont HL, et al Fecal microbiota transplantation for refractory immune checkpoint inhibitor-associated colitis. Nat Med. 2018;24(12):1804–8. 10.1038/s41591-018-0238-9 30420754 PMC6322556

[45] Kashem SW, Igyarto BZ, Gerami-Nejad M, Kumamoto Y, Mohammed JA, Jarrett E, et al *Candida albicans* morphology and dendritic cell subsets determine T helper cell differentiation. Immunity. 2015;42(2):356–66. 10.1016/j.immuni.2015.01.008 25680275 PMC4343045

[46] Shao TY, Ang WXG, Jiang TT, Huang FS, Andersen H, Kinder JM, et al Commensal *Candida albicans* Positively Calibrates Systemic Th17 Immunological Responses. Cell Host Microbe. 2019;25(3):404–17 e6. 10.1016/j.chom.2019.02.004 30870622 PMC6419754

[47] Bacher P, Hohnstein T, Beerbaum E, Rocker M, Blango MG, Kaufmann S, et al Human Anti-fungal Th17 Immunity and Pathology Rely on Cross-Reactivity against *Candida albicans*. Cell. 2019;176(6):1340–55 e15. 10.1016/j.cell.2019.01.041 30799037

[48] Bacher P, Kniemeyer O, Schonbrunn A, Sawitzki B, Assenmacher M, Rietschel E, et al Antigen-specific expansion of human regulatory T cells as a major tolerance mechanism against mucosal fungi. Mucosal Immunol. 2014;7(4):916–28. 10.1038/mi.2013.107 24301658

[49] Rizzetto L, Kuka M, De Filippo C, Cambi A, Netea MG, Beltrame L, et al Differential IL-17 production and mannan recognition contribute to fungal pathogenicity and commensalism. J Immunol. 2010;184(8):4258–68. 10.4049/jimmunol.0902972 20228201

[50] Kesavan S, Walters CE, Holland KT, Ingham E. The effects of *Malassezia* on pro-inflammatory cytokine production by human peripheral blood mononuclear cells in vitro. Med Mycol. 1998;36(2):97–106. 9776820

[51] Strati F, Calabro A, Donati C, De Felice C, Hayek J, Jousson O, et al Intestinal *Candida parapsilosis* isolates from Rett syndrome subjects bear potential virulent traits and capacity to persist within the host. BMC Gastroenterol. 2018;18(1):57 10.1186/s12876-018-0785-z 29720131 PMC5930502

[52] Tso GHW, Reales-Calderon JA, Tan ASM, Sem X, Le GTT, Tan TG, et al Experimental evolution of a fungal pathogen into a gut symbiont. Science. 2018;362(6414):589–95. 10.1126/science.aat0537 30385579

[53] Quintin J, Saeed S, Martens JHA, Giamarellos-Bourboulis EJ, Ifrim DC, Logie C, et al *Candida albicans* infection affords protection against reinfection via functional reprogramming of monocytes. Cell Host Microbe. 2012;12(2):223–32. 10.1016/j.chom.2012.06.006 22901542 PMC3864037

[54] Rizzetto L, Ifrim DC, Moretti S, Tocci N, Cheng SC, Quintin J, et al Fungal Chitin Induces Trained Immunity in Human Monocytes during Cross-talk of the Host with *Saccharomyces cerevisiae*. J Biol Chem. 2016;291(15):7961–72. 10.1074/jbc.M115.699645 26887946 PMC4825003

[55] Angebault C, Ghozlane A, Volant S, Botterel F, d'Enfert C, Bougnoux ME. Combined bacterial and fungal intestinal microbiota analyses: Impact of storage conditions and DNA extraction protocols. PLoS ONE. 2018;13(8):e0201174 10.1371/journal.pone.0201174 30074988 PMC6075747

[56] Frau A, Kenny JG, Lenzi L, Campbell BJ, Ijaz UZ, Duckworth CA, et al DNA extraction and amplicon production strategies deeply inf luence the outcome of gut mycobiome studies. Sci Rep. 2019;9(1):9328 10.1038/s41598-019-44974-x 31249384 PMC6597572

[57] Nilsson RH, Ryberg M, Kristiansson E, Abarenkov K, Larsson KH, Koljalg U. Taxonomic reliability of DNA sequences in public sequence databases: a fungal perspective. PLoS ONE. 2006;1:e59 10.1371/journal.pone.0000059 17183689 PMC1762357

[58] Tang J, Iliev ID, Brown J, Underhill DM, Funari VA. Mycobiome: Approaches to analysis of intestinal fungi. J Immunol Methods. 2015;421:112–21. 10.1016/j.jim.2015.04.004 25891793 PMC4451377

[59] Wingard JR. Have novel serum markers supplanted tissue diagnosis for invasive fungal infections in acute leukemia and transplantation? Best Pract Res Clin Haematol. 2012;25(4):487–91. 10.1016/j.beha.2012.10.013 23200547 PMC3513695

[60] Kozel TR, Wickes B. Fungal diagnostics. Cold Spring Harb Perspect Med. 2014;4(4):a019299 P 10.1101/cshperspect.a019299 24692193 PMC3968782

[61] Zhai B, Ola M, Rolling T, Tosini NL, Joshowitz S, Littmann ER, et al High-resolution mycobiota analysis reveals dynamic intestinal translocation preceding invasive candidiasis. Nat Med. 2020 10.1038/s41591-019-0709-7 PMC7005909 31907459

[62] Shelburne SA, Ajami NJ, Chibucos MC, Beird HC, Tarrand J, Galloway-Pena J, et al Implementation of a Pan-Genomic Approach to Investigate Holobiont-Infecting Microbe Interaction: A Case Report of a Leukemic Patient with Invasive Mucormycosis. PLoS ONE. 2015;10(11):e0139851 10.1371/journal.pone.0139851 26556047 PMC4640583

[63] van Leeuwen PT, van der Peet JM, Bikker FJ, Hoogenkamp MA, Oliveira Paiva AM, Kostidis S, et al Interspecies Interactions between *Clostridium difficile* and *Candida albicans*. mSphere. 2016;1(6). 10.1128/mSphere.00187-16 PMC5103046 27840850

[64] Panpetch W, Somboonna N, Palasuk M, Hiengrach P, Finkelman M, Tumwasorn S, et al Oral *Candida* administration in a *Clostridium difficile* mouse model worsens disease severity but is attenuated by Bifidobacterium. PLoS ONE. 2019;14(1):e0210798 10.1371/journal.pone.0210798 30645630 PMC6333342

